# "Healthy Anorexia": rationalising contradictions

**DOI:** 10.1186/2050-2974-1-S1-O67

**Published:** 2013-11-14

**Authors:** Connie Musolino, Megan Warin, Tracey Wade, Peter Gilchrist

**Affiliations:** 1 Gender Studies and Social Analysis, University of Adelaide, Australia; 2 School of Psychology, Flinders University, Australia; 3 Weight Disorder Unit, Flinders Medical Centre, Australia

## 

This paper explores how young women with disordered eating rationalise their behaviour as 'healthy'. Based on preliminary findings from an Australian Research Council grant that is investigating why people with eating disorders are reluctant to engage with treatment services, we demonstrate how young women use normative discourses of body surveillance, 'healthy eating' and self-discipline to maintain and support eating disorder practices. Healthy lifestyles are supported by a range of public health initiatives that promote 'watching one's weight', taking regular exercise and eating foods that are low in fat. Such culturally sanctioned discourses are readily available for people with eating disorders to position themselves within, providing a normative space to practice body surveillance, and also a legitimate and moral claim to looking after oneself. Investigating the parameters in which people rationalise excessive dietary restriction, and over-exercising as a healthy lifestyle is important for early detection of eating disorders, and to the development of strategies that challenge the ease in which eating disorders can be hidden in everyday health practices.

This abstract was presented in the **Understanding and Treating Eating Pathology** stream of the 2013 ANZAED Conference.

